# Testicular Torsion as a Complication Following Preperitoneal Packing for Pelvic Fractures in a Motorcycle Accident: A Case Report

**DOI:** 10.7759/cureus.64996

**Published:** 2024-07-20

**Authors:** Thomas Caussat, Hayley Kong, Brandon Macknofsky, Adam Mann, Mario Rueda, Robert Borrego

**Affiliations:** 1 Psychiatry, Florida Atlantic University Charles E. Schmidt College of Medicine, Boca Raton, USA; 2 Orthopedic Surgery, Florida Atlantic University Charles E. Schmidt College of Medicine, Boca Raton, USA; 3 Surgery, Florida Atlantic University Charles E. Schmidt College of Medicine, Boca Raton, USA; 4 Trauma, Florida Atlantic University/St. Mary’s Medical Center, West Palm Beach, USA; 5 Surgery, St. Mary's Medical Center, West Palm Beach, USA

**Keywords:** preperitoneal packing, multidisciplinary approach, postoperative complications, urological emergencies, trauma surgery, pelvic fractures, testicular torsion

## Abstract

This case report highlights a rare but significant complication of blunt trauma requiring preperitoneal packing and illustrates the intricate relationship between trauma surgery and urological emergencies. Testicular torsion is an acute urological emergency necessitating prompt surgical intervention to salvage testicular function. While commonly associated with intrinsic factors such as the "bell-clapper" deformity, extrinsic factors such as trauma and postoperative complications can also precipitate this condition. This case underscores the complexity of diagnosing and managing testicular torsion arising after surgical interventions for pelvic fractures, a scenario sparsely documented in medical literature. We present a 27-year-old male who sustained multiple injuries, including a pelvic fracture, from a motorcycle accident and subsequently underwent preperitoneal packing for significant pelvic hemorrhage. Five days post-operation, the patient developed acute right lower quadrant and unilateral testicular pain, leading to the diagnosis of testicular torsion via Doppler ultrasonography. An emergency bilateral orchiopexy was performed, revealing a 180° torsion of the right testis. This case illustrates the need for a heightened awareness of potential genitourinary complications following trauma surgery. The pathophysiological mechanisms possibly include increased intra-abdominal pressure and altered testicular mobility due to surgical interventions. The report emphasizes the importance of multidisciplinary care in trauma settings to ensure comprehensive evaluation and management of patients, including the consideration of urological complications. Testicular torsion following preperitoneal packing for pelvic fractures represents a critical intersection between trauma surgery complications and urological emergencies, necessitating vigilant postoperative care and multidisciplinary collaboration for timely diagnosis and intervention. This case contributes to the broader understanding of postoperative complications, advocating for an integrated approach to patient care in high-energy trauma scenarios.

## Introduction

Testicular torsion following preperitoneal packing is an underreported complication in the adult population. Testicular torsion, a critical urologic emergency characterized by twisting of the spermatic cord leading to compromised blood flow to the testis, demands prompt recognition and surgical intervention to prevent irreversible testicular ischemia and subsequent loss [1]. While intrinsic anatomical abnormalities such as the "bell-clapper" deformity are frequent culprits, extrinsic factors such as trauma can precipitate this condition as well. Although current literature shows testicular dislocation secondary to blunt scrotal trauma [2], pelvic ring injuries (particularly following motorcycle accidents [3-7]), and torsion secondary to cryptorchidism in the pediatric population [8], the association between pelvic surgery and subsequent testicular torsion in adults is not well documented. This case of testicular torsion following preperitoneal packing is significant for its rarity as well as the insight it maps along the complex interplay of surgical intervention and testicular health.

## Case presentation

We report the case of a 27-year-old male with no significant medical or surgical history who was involved in a motorcycle accident and ejected from the bike. Upon presentation, he was hemodynamically stable, alert, and oriented, with a Glasgow Coma Case (GCS) of 15. The physical exam was notable for an obvious laceration to his glabella, bruises over his face, right hip, and right wrist, and minor lacerations diffusely. An obvious deformity to his right leg with external rotation was noted, with intact distal pulses bilaterally. While bruising and ecchymoses were found diffusely across the lower abdomen, pelvis, upper back, and bilateral upper and lower extremities, no significant ecchymosis, bruising, or swelling was reported overlying the testicles or perineum. The pelvis was found to be unstable. X-rays of the chest, pelvis, and left upper extremity were performed in the trauma bay, which showed an obvious open-book pelvic fracture and a left wrist dislocation. Initial management with a pelvic binder and a wrist splint showed radiographic improvement in pelvic reduction and wrist alignment.

**Figure 1:**
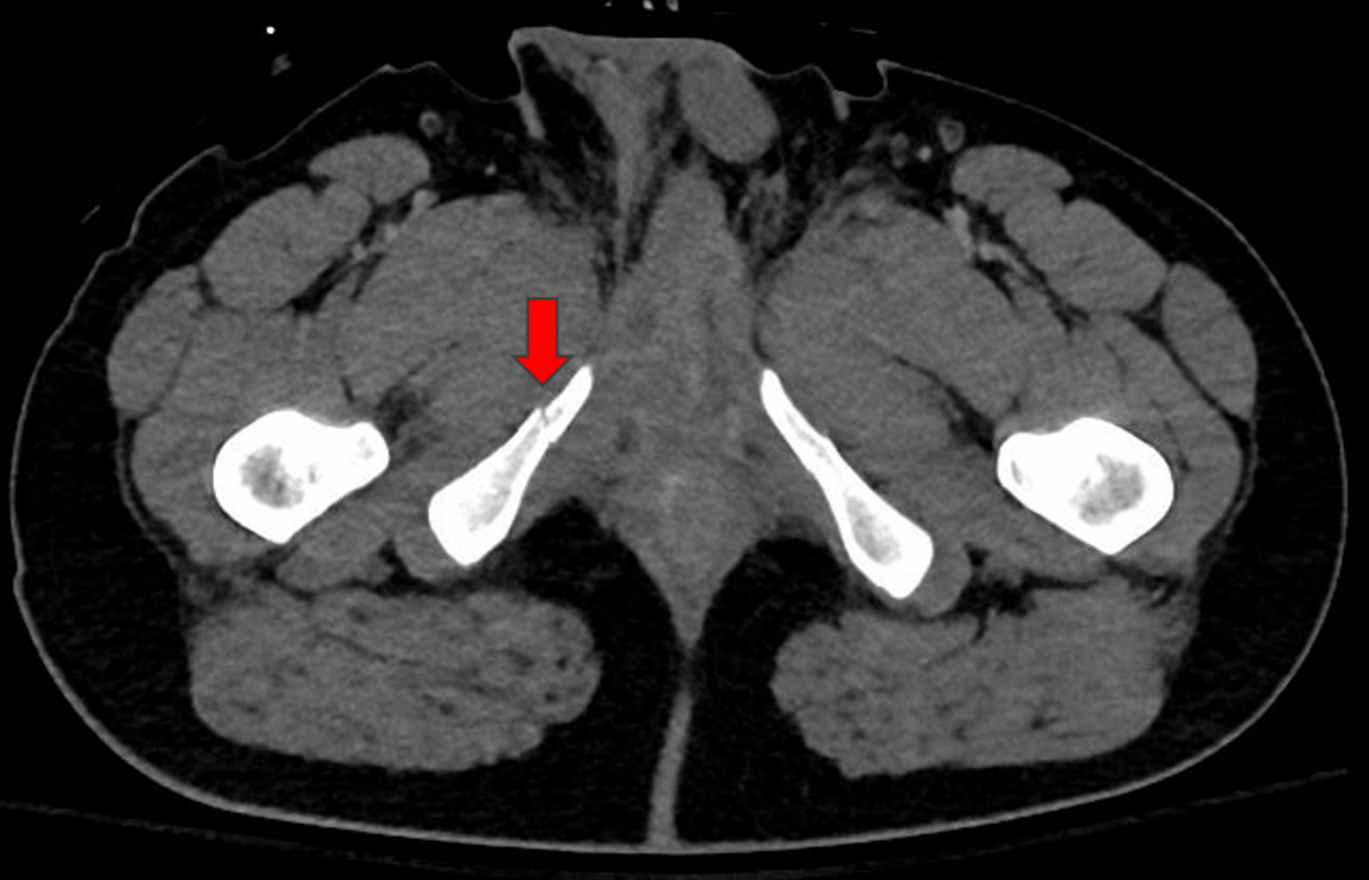
CT abdomen/pelvis demonstrating nondisplaced pubic fracture (red arrow). CT: computed tomography

**Figure 2:**
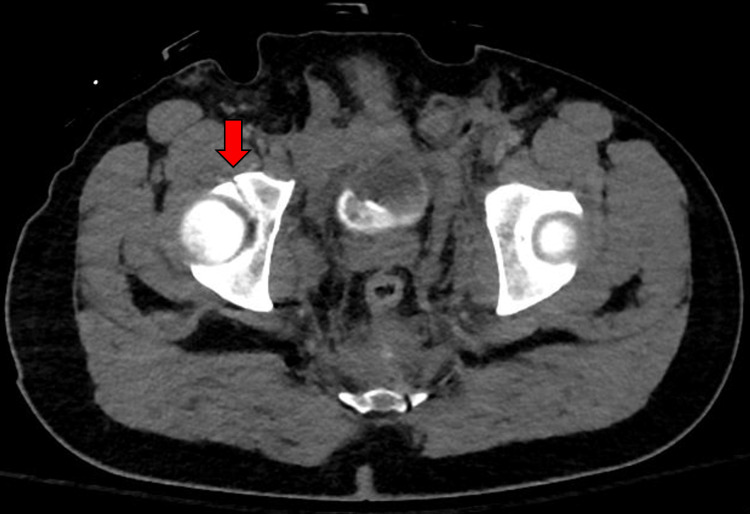
CT abdomen/pelvis demonstrating nondisplaced fractures of the right acetabulum (red arrow). CT: computed tomography

A focused assessment with sonography in trauma (FAST) exam was negative for intra-abdominal bleeding, and a retrograde urethrogram ruled out urethral injury. Following initial stabilization, computed tomography (CT) and lower extremity X-rays were performed, revealing a marked diastasis of the symphysis pubis and left sacroiliac joint, right obturator ring fractures, a right acetabular fracture, and an associated large pelvic hemorrhage with active bleeding in the presacral region. Non-pelvic injuries include a dislocation of the left wrist associated with a soft tissue injury (Figures 1, 2).

Due to the significant pelvic bleeding identified on CT, the patient underwent emergent preperitoneal pelvic packing. The bladder was palpated and intact. Pelvic fractures were palpable in the pelvic ring; 1 L of blood was evacuated from the preperitoneal space, seven lap pads were placed, and the patient was admitted to the ICU for monitoring. The packing was removed two days later without complication. A 10 French Jackson-Pratt drain (Cardinal Health, Dublin, Ohio) was placed in the preperitoneal space, after which the orthopedic team performed an open reduction and internal fixation of the pelvis.

**Figure 3:**
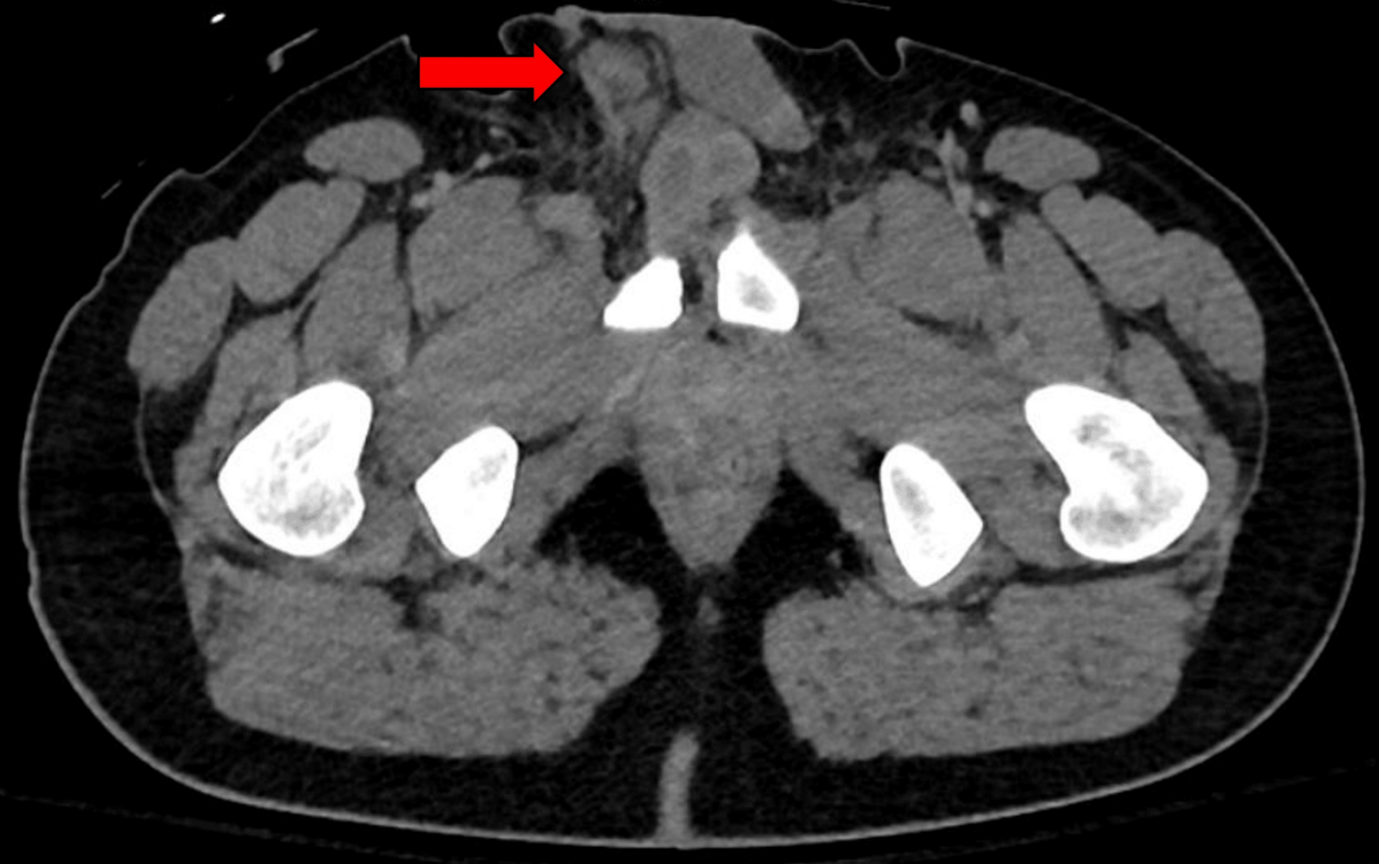
CT pelvis demonstrating an incidental finding of the right testis elevated in the inguinal canal (red arrow). CT: computed tomography

**Figure 4:**
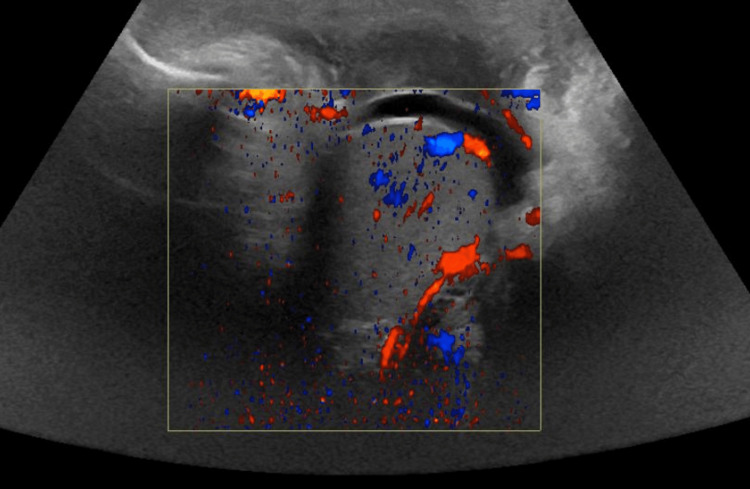
Reduced blood flow to the right testicle on Doppler ultrasound.

Five days following preperitoneal packing, the patient reported acute-onset right lower quadrant (RLQ) abdominal pain and unilateral testicular pain. The right testicle was difficult to palpate on physical exam and higher in the inguinal canal. CT scans taken during admission were reviewed and revealed a high-riding right testicle (Figure 3). Urology was contacted emergently, and a Doppler ultrasonography revealed that the right testicle measured 4.4 x 2.7 x 2.3 cm and demonstrated grossly unremarkable echotexture without arterial flow, suspicious for testicular torsion (Figure 4). The left testis was measured at 3.8 x 2.2 x 2.6 cm with normal color flow and echotexture.

An emergency bilateral orchiopexy was performed, revealing a 180° torsion of the right testis high in the distal inguinal canal. Orchiopexy of the left testicle was also performed to reduce the chance of contralateral torsion. Further history obtained from the patient following emergent orchiopexy revealed no prior history of testicular trauma, genitourinary complications in childhood, congenital abnormalities, inguinal hernias, or known undescended testes. The patient has no prior abdominal or pelvic imaging that could have been used as a pre-trauma comparison.

After several days of multidisciplinary follow-up, the patient was admitted to inpatient rehab, where he regained strength and functionality. He did not report any new pain, did not require any additional blood products, and denied any urological symptoms or sexual dysfunction prior to discharge following 10 days of rehabilitation.

## Discussion

The development of postoperative testicular torsion following preperitoneal packing presents a unique intersection of trauma surgery complications and acute urological emergencies. The pathophysiological basis for this occurrence may involve several factors. While not read on the first impression, the high-riding testicle was present on the initial CT scan, making it possible that this torsion was a result of the initial trauma, although this is less likely given that he did not have pain for several days leading up to his acute RLQ pain. Additionally, the patient is young and has no known medical history, including any history of urogenital abnormalities in childhood, prior testicular trauma, inguinal hernias, abdominal surgeries, or known undescended testes. Notably, as the patient had no abdominal/pelvic imaging prior to this accident, it cannot be definitively known if the high-riding testicle occurred secondary to this trauma or if it was simply previously undiagnosed. However, given the timing of the acute-onset RLQ pain on hospital day five after the initial trauma, we believe that this patient’s torsion is likely secondary to the pelvic packing he received on hospital day one. The increase in intra-abdominal pressure secondary to preperitoneal packing could have indirectly affected the spermatic cord and promoted torsion, with or without a preexisting anatomical abnormality. Additionally, the displacement or manipulation of internal structures during preperitoneal packing may have contributed to altered testicular mobility, increasing the risk of torsion.

Although, to our knowledge, there have been no documented cases of torsion induced by preperitoneal packing, there are several reports of testicular injury following pelvic fractures and blunt scrotal trauma, particularly following motorcycle accidents [3-7]. Our patient’s genitourinary complication was identified five days following the initial emergent intervention, similar to a case reported by Bernhard et al. [3], wherein testicular dislocation was discovered during pelvic stabilization surgery three days post-trauma. The delay in recognizing testicular torsion or dislocation, despite the patient's early and aggressive management of pelvic injuries, highlights the critical need for ongoing evaluation and consideration of genitourinary complications in the aftermath of pelvic trauma. Similarly, Bernhard et al. [3] highlight the essential role of a multidisciplinary approach in trauma care, emphasizing not only the surgical intervention but also the meticulous postoperative monitoring for complications for an extended duration, as the onset of complications may vary between patients. In our case, the patient's acute-onset RLQ and testicular pain prompted an urgent Doppler ultrasonography on post-packing day five, leading to the diagnosis of compromised arterial flow to the right testis, suggestive of testicular torsion.

Similar to our patient, Wiznia et al. [4] reported a case of testicular dislocation into the inguinal canal associated with a lateral compression-type pelvic ring injury secondary to a motorcycle accident. However, their case did not involve preperitoneal packing or delayed testicular torsion. A similar report by Boudisa et al. [6] highlighted a case of displacement of both testicles and immediate emergency bilateral orchiopexy without subsequent gonadal dysfunction. Their early recognition of ectopy through CT imaging and prompt surgical identification and correction likely reduced the chances of developing testicular torsion. Of note, preperitoneal packing was again not part of their treatment prior to orchiopexy. Chiu and Lin [5] reported a pelvic injury and testis rupture secondary to motorcycle trauma, which led to radical orchiectomy. Similarly, Lenfant et al. [7] published a case of pubic testicular dislocation by the same mechanism, again managed with unilateral orchiectomy.

These reports all align with the same general recommendations: advocating for diligent pre- and intraoperative assessments to prevent missed diagnoses of testicular dislocation or torsion, which can be easily overlooked in the setting of severe injuries such as pelvic fractures, long bone fractures, or intra-abdominal bleeding. They highlight the necessity for trauma and orthopedic teams to remain alert to the possibility of genitourinary complications throughout a patient’s hospital stay, even when initial evaluations such as FAST exams and retrograde urethrograms do not indicate intra-abdominal or urethral injuries. Rapid diagnosis and treatment are key to reducing the likelihood of long-term complications such as testicular ischemia, testicular atrophy, chronic discomfort, and infertility [9,10].

## Conclusions

This case of testicular torsion following preperitoneal packing for pelvic fractures highlights a rare but grave complication, emphasizing the critical necessity for a multidisciplinary approach that integrates trauma and urological surgical expertise. It underscores the importance of vigilance in postoperative care, particularly for symptoms that may not align with the primary focus of the surgery, and serves as a pivotal reminder of the potential for unexpected complications. This contribution to the medical literature delineates the significance of prompt recognition and intervention in managing postoperative complications, advocating for comprehensive patient care that spans multiple specialties. The case underlines the need for further studies and case reports to elucidate the mechanisms behind such complications and to establish guidelines for their prevention and management. By sharing our experience and highlighting areas for improvement, this report aims to enhance the understanding and management of potential sequelae following trauma surgery, thereby raising awareness and contributing to improved patient outcomes in similar high-energy trauma scenarios.
